# Green Investment Changes in China: A Shift-Share Analysis

**DOI:** 10.3390/ijerph18126658

**Published:** 2021-06-21

**Authors:** Ruxu Sheng, Rong Zhou, Ying Zhang, Zidi Wang

**Affiliations:** 1 School of Public Policy and Management, Tsinghua University, Beijing 100084, China; shengruxv@mail.tsinghua.edu.cn (R.S.); wangzd20@mails.tsinghua.edu.cn (Z.W.); 2 International Business School, Brandeis University, Waltham, MA 02453, USA; yz95youth@163.com

**Keywords:** green investment, regional analysis, homothetic shift-share analysis, driving factor

## Abstract

As China’s economic development has entered a new phase, China needs to seek a new path of green transformation development to coordinate the economic growth with environmental mitigation. From 2002 to 2017, green investment in China grew from 118.56 billion Chinese yuan to 950.86 billion Chinese yuan, increasing more than seven times. In this study, a homothetic shift-share analysis (HSSA) is used to understand how green investment changed and was used to decompose the change of provincial green investment in China from 2002 to 2017 into four driving factors: the national economic growth effect (*NEG*), national green investment structure effect (*NIS*), homothetic regional green investment competition effect (*HRIC*), and regional green investment allocation effect (*RIA*). The results indicate that these four factors had various regional and temporal characteristics, although green investment increased in all provinces during this period. More specifically, the *NEG* was more significant in the east than in other regions. The regional differences of *NEG* were relatively large in the first two periods (2002–2007 and 2007–2012) and began to shrink in the third period (2012–2017). The *NIS* shared the same characteristics as the *NEG*. In terms of *HRIC*, the central region was ahead of the eastern and western regions, and relatively many eastern provinces were with negative *HRIC*. The *HRIC* of most provinces showed a trend of “low/medium-medium/high-low”. The *RIA* inhibited green investment growth in most provinces and showed a “high-low-high” trend regarding the change from 2002 to 2017. Our study suggests that it is necessary to coordinate the growth of green investment across different regions and establish an ecological compensation mechanism.

## 1. Introduction

China has witnessed an almost unprecedented continuous high economic growth during the last 30 years and has consumed a large amount of energy and caused serious environmental degradation [1], causing China to be regarded and criticized as a reckless growth-at-all-costs polluter by developed countries over recent decades. Many studies have also focused on the negative impact caused by China’s economic and industrial development while not paying enough attention to the huge input and contribution made by China in environmental mitigation. With rapid economic and industrial modernization, there is no mono causal-relationship between economic development and the environment [2]. On the one hand, economic and industrial development increases resources extraction and consumption and relates to pollutant emissions, resulting in greater environmental pressure [3]. On the other hand, the growing productivity offers more possibilities for green investment [4], green technology and equipment upgrading [5,6,7], and even sophisticated environmental governance capacity. To better understand the current state of the environment, we should interpret both of these contrasting tendencies in China.

Unlike some countries’ retreat from the global environmental governance regime, China has become a leader in environmental actions in the last few years [8,9]. Since the 11th Five-Year Plan, the Chinese government has taken the leadership in tightening environmental governance [10]. A lot of direct government investment has been made on the public infrastructure to improve energy saving and create a favorable market environment for private green economy development [11]. Meanwhile, with environmental regulations tightening, private firms are also required to invest more in anti-pollution [12]. Against this background, China has become the leader in global growth in green investment, especially in the energy sector [4,13,14]. Some scholars have praised China as a “green entrepreneurial state” because of the critical effort in green transformation [15].

Green investment is the foundation and important determinant for environmental governance. Firstly, massive green investment is necessary to develop environmental protection projects, which are characterized by a long investment cycle and low return [16]. Secondly, considering the positive knowledge externalities of green technologies and equipment, private investments in green technologies are usually scarce, needing public support to compensate [17]. Thirdly, the growth of green investment indicates that there exist potential opportunities for firms that conduct cleaner production, which will guide the productive behaviors of firms. Lastly, government interventions, such as implementing environmental taxes/charges and establishing an emission trading scheme, also demand massive green investments [18]. To better guide the next phase of environmental governance in China, we need to investigate the geographical distribution of China’s green investment across the country and understand the driving factors.

To understand how green investment changed, we decompose the change of provincial green investment in China during 2002–2017 into four factors: national economic growth effect, national green investment structure effect, homothetic regional green investment competition effect, and regional green investment allocation effect. Based on the decomposition results, we also discuss the differences in the time trends of provincial green investment drivers in the eastern, central and western parts of China. To the best of our knowledge, this study is the first to use shift-share analysis to analyze the driving factors of green investment changes in China. This study is considered as a limited marginal contribution to the research of green investment, and it is expected to provide valuable references for policy-makers in China to formulate the next step of environmental policy.

The article is organized as follows. Section 2 reviews the previous work. Section 3 introduces the data acquisition and the shift-share decomposition approach. Section 4 presents the analysis and discussion of the results. Conclusions are presented in Section 5.

## 2. Literature Review

### 2.1. Green Investment

Along with the extensive focus on climate change, green investment has also attracted more attention from academic scholars and policy-makers. Broadly speaking, it refers to social investments used for supporting environmental improvement, also known as environmental investments [18]. It includes investments in infrastructure, industrial pollution treatment and environmental facilities of projects. Some previous research concentrated on analyzing determinant factors that affect green investment growth. Eyraud et al. empirically investigated the macroeconomic drivers of green investment, and they found that economic growth, a sound financial system conducive to low interest rates, high fuel prices and some policy interventions, including carbon pricing schemes and “feed-in-tariffs”, are key boosters to green investment [4]. Murovec et al. focused on the same question but from a microeconomics perspective [19]. Apart from policy measures, they found that past environmental investments, the importance of environmental technologies for customers and the firm performance have a positive effect on the environmental investments of firms. On the contrary, research about marine renewable energy investment in the UK indicated that policy instability, level of capital and revenue support are key barriers to green investment [20].

As for green investment in China, its development is in line with the development trend of the world. In response to environmental issues, countries worldwide have been using green investments to fund the transition to more sustainable economies. Besides, green investments in China have their own characteristics. Firstly, while gross domestic product growth has driven up the green investment, the amount of green investment in China has increased dramatically but still cannot meet the demand. However, this growth rate is hardly sufficient to meet the needs of large-scale transformation. Secondly, the development of green investments in 31 Chinese provinces varies greatly [18]. Environmental degradation and climate change are global phenomena with marked local manifestations [21]. The target and demand for green investment in different places are vastly different, and the level of economic development in different regions also affects the development of the green investment. Thirdly, the development of green investment in China is highly dependent on the government. As the green finance market has not been fully established in China, the development of green investment needs to be driven and supported by policies.

To investigate the influencing factors of green investments, Du et al. empirically tested the influence from economic factors, environmental factors and political factors [18]. They found that, in China, the economic and environmental factors play more significant roles than political factors do. The economic factors containing GDP, GDP per capita and fixed assets investment are the basis of GI development. The environmental factors, including wastewater discharge, emissions of waste gas and solid, also have a strong capability for driving the development. The political factor exists one-year time-lag effect, and the spillover effects of political factor are more significant than that of direct effects. From a microeconomics perspective, Zhu et al. used a duopoly competition game mode to study the influencing factors of a manufacturer’s green investment decision [22]. They found that the equilibrium decision-making of green investment is affected by the adjustment rate and cost of green investment. Xu et al. conducted a cross-industry study, and they found that policies had no influence on investment in the agriculture sector nor the construction sector but had a positive effect on investment in the transportation sector [23].

### 2.2. Shift-Share Analysis

Shift-share analysis (SSA) is a long-established and widely used index decomposition analysis in the regional study. The classical three-component SSA was first formalized formally by Dunn [24]. The model decomposed regional employment growth into three components: the national growth effect, the industry mix effect, and the competition effect. Due to the existence of interwoven effect in the second and third components, scholars have engaged in a protracted discussion on the component independence of classical SSA [25,26,27,28,29,30]. The most famous one is the homothetic SSA (also known as the E-M model), in which Esteban-Marquillas [27] creatively introduced “homothetic variables” to deal with component independence. For details of discussion on component independence and the development of various models, one can turn to Loveridge and Selting [31]. A brief mathematical explanation is introduced in the methodology section of this study.

Originally, SSA was intended to analyze regional industrial development based on employment [24], but now SSA has been widely applied in many new fields, including the environmental and energy fields, such as the development of financial inclusion [32], development of cooperative societies [33], rural economic development [34], dynamics of the population ageing process [35], etc. In the field of environment and energy, SSA involved two main types of applications. The first type analyzed the impact of regional industrial structure changes on the regional environment. This type of research still used SSA in industrial analysis, but mainly for industries related to the environment and energy. Zheng et al. used SSA to measure the industrial restructuring in China. Then they investigated the impact of industrial restructuring on the level of provincial green development from the perspective of mineral resource security [36]. Liu and Dong used SSA to measure the transformation of China’s provincial industrial manufacturing industries. Then the influence of the industrial transformation process on haze pollution in China was discussed [37]. The second type used SSA to decompose changes in indicators related to environment and energy. This type of research was no longer limited to industry analysis but used SSA to explore the driving factors of change in environment and energy characteristics. Blanco et al. used SSA to analyze the use of three main renewable energies, which are hydro, wind and solar photovoltaic, in Spain. Based on this, the Renewable Energy Plan 2011–2020 was evaluated [38]. Gilli et al. used SSA to analyze the variation of emission intensity in manufacturing sectors. Then the driving factors and decoupling trends of environmental pressures generated by manufacturing production and consumption in selected developed and developing countries were assessed [39]. This type of research also applied SSA directly to energy and electricity consumption studies, such as energy demand change in Japan [40], electricity consumption changes in Italian [41], electricity intensities in Italy [42], electricity consumption changes in China [43], energy consumption changes in China [44], etc.

On the basis of the above studies, we summarize the characteristics as follow. Firstly, many scholars recognized that the development of green investment was closely related to economic development but also influenced by other factors, including local environment and government intervention. Secondly, many studies focused on using SSA to analyze the drivers of regional energy consumption. Few studies had discussed drivers of environmental variables, let alone explore the driving factors of green investments. Meanwhile, these studies did not pay attention to the regional differences in green investment development. As mentioned earlier, there are obvious regional differences in the manifestation of environmental degradation, and different regions have different economic and policy situations. These factors determine the uneven distribution and development of China’s green investment, which needs further attention. Therefore, considering the continuous rapid growth of green investment in China over the past decade and the regional differences, it is of great significance to study the driving mechanism and provincial distribution of green investment in China.

## 3. Methodology and Data

### 3.1. Shift-Share Analysis and Its Homothetic Model Extension

This study aims to analyze drivers of change in provincial green investment in China. To introduce our methodology, homothetic Shift-share analysis (HSSA), we follow classical Shift-share analysis (SSA) and include some notation. Let $g_{i}$ stand for the rate of change of green investment in province i, decomposed by using the formula:

(1) $$g_{i}=y+\left(g_{0}-y\right)+\left(g_{i}-g_{0}\right),$$

where $g_{0}$ and y are the rates of change of national green investment and rate of change of national GDP, respectively. $GI_{i}$ being the green investment in province i, the decomposition in Equation (1) can be reformulated as follows:

(2) $$\Delta GI_{i}=GI_{i}\times g_{i}=GI_{i}\times y+GI_{i}\times \left(g_{0}-y\right)+GI_{i}\times \left(g_{i}-g_{0}\right),$$

where $GI_{i}$, rate of change of green investment in province i, is decomposed into three components. In Equation (2), the National economic growth effect (hereafter, *NEG*) is $GI_{i}\times y$ and indicates the amount of change in the green investment of province i, if the province changes at the rate of change of national GDP.

The National Green Investment Structure Effect (henceforth, *NIS*) is $GI_{i}\times \left(g_{0}-y\right)$ and represents the regional effect of the difference between the national green investment growth rate and the national GDP growth rate. More specifically, if the national green investment grows faster than the national GDP does, which means $g_{0}-y$ > 0, the change in green investment in province i due to the national structure effect is positive.

The third part is the Regional Green Investment Competition Effect (henceforth, *RIC*), measured by $GI_{i}\times \left(g_{i}-g_{0}\right)$ and reflects the green investment competitiveness of province i within all provinces in China. A positive (negative) value of *RIC* indicates a faster (slower) growth of green investment of province i than that of national green investment.

The discussion of the property of the component independence promoted the subsequent development of classical SSA. Esteban-Marquillas [27] introduced “homothetic variables” into SSA and proposed the homothetic shift-share analysis (HSSA) to ensure the component independence of the third component, *RIC*. This part can be reformulated as follows:

(3) $$RIC_{i}=GI_{i}\times \left(g_{i}-g_{0}\right)=Y_{i}\times GI_{i}/Y_{i}\times \left(g_{i}-g_{0}\right),$$

where $Y_{i}$ is the GDP of province i, while $GI_{i}/Y_{i}$ is considered as the share of green investment in GDP of province i. Concluding from Equations (2) and (3), both *RIC* and *NIS* are dependent and influenced by the same factor, structure, which suggests there exist issues of the property of the component independence. This means that *RIC* cannot be considered as a pure competition effect. To deal with this problem, we introduce the “homothetic variable”. Let $GI_{i}^{H}$ be the “homothetic green investment”, calculated by the formula followed:

(4) $$GI_{i}^{H}=Y_{i}\times GI_{0}/Y_{0},$$

where $GI_{0}$ and $Y_{0}$ are national green investment and GDP, respectively, while $GI_{0}/Y_{0}$ is considered as the national share of green investment in GDP. Assume that the green investment of province i is determined by the product of the provincial GDP and the national green investment share. We obtain the homothetic variable, $GI_{i}^{H}$, as shown in Equation (4). Now, the following variables are defined:

(5) $$RIC_{i}=HRIC_{i}+RIA_{i},$$

(6) $$HRIC_{i}=GI_{i}^{H}\times \left(g_{i}-g_{0}\right),$$

(7) $$RIA_{i}=\left(GI_{i}-GI_{i}^{H}\right)\times \left(g_{i}-g_{0}\right),$$

In Equation (5), the Regional Green Investment Competition Effect, *RIC*, is decomposed into two components. The Homothetic Regional Green Investment Competition Effect (hereafter, *HRIC*) in Equation (6) reflects the purer competition effect of excluding the regional industrial structure.

The Regional Green Investment Allocation Effect (henceforth, *RIA*) in Equation (7) is multiplied by two differences and reflects whether the province is specializing in advantageous green investment or not. More specifically, if the province has a comparative advantage on growth $\left(g_{i}-g_{0}>0\right)$ and it focuses more on green investment than the entire country $\left(GI_{i}-GI_{i}^{H}>0\right)$, the *RIA* is positive.

By introducing homothetic green investment, we decompose the provincial changes of green investment into four components using HSSA, and it is calculated by the formula followed:

(8) $$\Delta GI_{i}=GI_{i}\times g_{i}=NIG_{i}+NIS_{i}+HRIC_{i}+RIA_{i},$$

where *NEG* and *NIS*, in Equation (8), represent the effect of national economic growth and national green investment structure on regional green investment change, respectively. *HRIC* represents the effect of provincial green investment competitiveness on green investment change, excluding the difference in green investment structure, and *RIA* indicates the extent of the distributional effect on green investment change, which is jointly determined by the degree of regional specialization and comparative advantage.

Meanwhile, to obtain the different changes of green investment in each province in each period, considering data availability and comparability across time, we use HSSA to study the changes of green investment in each province in three stages: 2002–2007, 2007–2012, and 2012–2017.

### 3.2. Data Sources of Green Investment in China

This study measures the level of green investment in China by using “investment in anti-pollution projects” of the China Bureau of Statistics, although various national government documents and studies have different definitions of green investment. There are three main reasons for this approach: (1) Considering the realistic background and the existing literature, we used investment in anti-pollution projects to measure green investment [18]. (2) Considering the availability of data, investment in anti-pollution projects in China and the various types of investments included in them have statistical data for a relatively long period. In contrast, most other qualitative studies’ definition of green investment cannot be accurately measured in quantitative studies. (3) Investment in anti-pollution projects is an indicator that the Chinese government highly values. On the one hand, investment in anti-pollution projects has been specifically counted in China Statistical Yearbook, China Environmental Statistical Yearbook, China Environmental Yearbook and any other authoritative yearbooks. On the other hand, when the China Bureau of Statistics compiled the China Statistical Yearbook, the authorities especially calculated “investment in anti-pollution projects as a percentage of GDP (%)”. This index’s importance can be seen from the fact that another indicator related to GDP in the China Statistical Yearbook is the ratio of R&D expenditure to GDP (%). In particular, the official document “National Urban Ecological Protection and Construction Plan (2015–2020)”, which was issued by the Ministry of Housing and Urban-Rural Development of China and Ministry of Ecology and Environment of China, uses “investment in anti-pollution projects as percentage of GDP (%)” as one of the important policy objectives. These three reasons ensure that investment in anti-pollution projects can be used in this study to reflect the level of green investment in China more accurately.

In China Statistical Yearbook in 2004, the China Bureau of Statistics gave a relatively complete definition and statistics of investment in anti-pollution projects for the first time. This definition has been used ever since. China Bureau of Statistics defines investment in anti-pollution projects as “fixed assets investment in the treatment of industrial pollution and in the construction of environment infrastructure facilities in cities and towns”. More specifically, investment in anti-pollution projects includes three types of investment: (i) investment in treatment of industrial pollution; (ii) environment protection investment in environment protection acceptance project in this year; (iii) investment in the construction of environment infrastructure facilities in cities and towns. The first two types, (i) and (ii), are related to industrial pollution, of which (i) in turn includes treatment of waste water, treatment of waste gas, treatment of solid waste, treatment of noise pollution and treatment of other pollution. The third type, (iii), includes gas supply, central heating, sewerage projects, gardening and greening, sanitation. The first two types, (i) and (ii), are green investments made by industrial firms. The third type (iii) is green investment from the government. Taking the data of 2017 as an example, the total green investment in China was 953.90 billion CNY. Among them, the first and second types of investment from industrial firms were 68.15 billion CNY, 277.17 billion CNY, respectively. The third type of green investment from the government was 608.57 billion CNY.

To analyze the change of provincial green investment in China from 2002 to 2017 using HSSA, we need to obtain the data of investment in anti-pollution projects, GDPs, and corresponding rates of change for 30 provinces in mainland China for all years. Xizang is not included in this study due to the unavailability of the data. The change rates of variables for each province are calculated using the ending and beginning data. The original data, 2002–2017 provincial GDP, are obtained from the China Bureau of Statistics database. 2003–2017 provincial investment in anti-pollution projects is obtained from the China Environment Statistical Yearbook for each year. The provincial investment in anti-pollution projects data for the year 2002 is obtained from the 2003 China Environmental Yearbook and 2003 China Urban Construction Statistical Yearbook.

Considering China’s vast territory and regional difference in economic development, in order to analyze the regional differences, we divide the 30 provinces into eastern, central, and western according to the regional classification criteria of the Chinese Bureau of Statistics for each province. The full names and abbreviations of the provinces included in the three regions are as follows:

(1) Eleven eastern provinces: Beijing (BJ), Tianjin (TJ), Hebei (HE), Liaoning (LN), Shanghai (SH), Jiangsu (JS), Zhejiang (ZJ), Fujian (FJ), Shandong (SD), Guangdong (GD), Hainan (HI).

(2) Eight central provinces: Shanxi (SX), Jilin (JL), Heilongjiang (HL), Anhui (AH), Jiangxi (JX), Henan (HA), Hubei (HB), Hunan (HN).

(3) Eleven western provinces: Inner Mongolia (NM), Guangxi (GX), Chongqing (CQ), Sichuan (SC), Guizhou (GZ), Yunnan (YN), Tibet (XZ), Shaanxi (SN), Gansu (GS), Qinghai (QH), Ningxia (NX).

In the rest of this study, we use abbreviations instead of the full names of provinces.

## 4. Results and Discussion

### 4.1. Change of Green Investment in China

**Figure 1:**
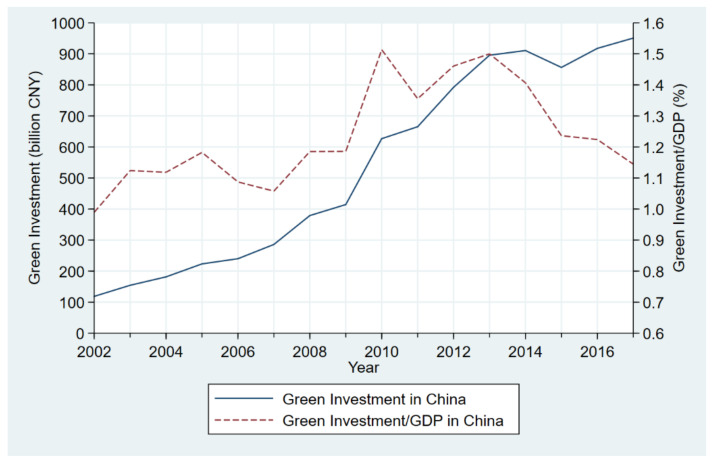
Green investment and green investment/GDP in China during 2002–2017.

Figure 1 shows the changes in green investment in China and green investment as a percentage of GDP in China from 2002–2017. Overall, green investment in China showed an upward sloping from 2002 to 2017, and green investment as a percentage of GDP in China maintained a steady growth trend. More specifically, green investment grew from 118.56 billion CNY in 2002 to 950.86 billion CNY in 2017, with an increase of 7.01 times during the period and an average annual growth rate of 14.89%. Although green investment increased in the general trend, the green investment as a percentage of GDP rose from a maximum of 0.99% in 2002 to 1.51% in 2010, as GDP also grew faster during the same period. Subsequently, it declined after 2013 and finally reached 1.14% in 2017. The green investment as a percentage of GDP increased by 0.15 percentage points during the period and was above 1.00% except for 2002.

**Figure 2:**
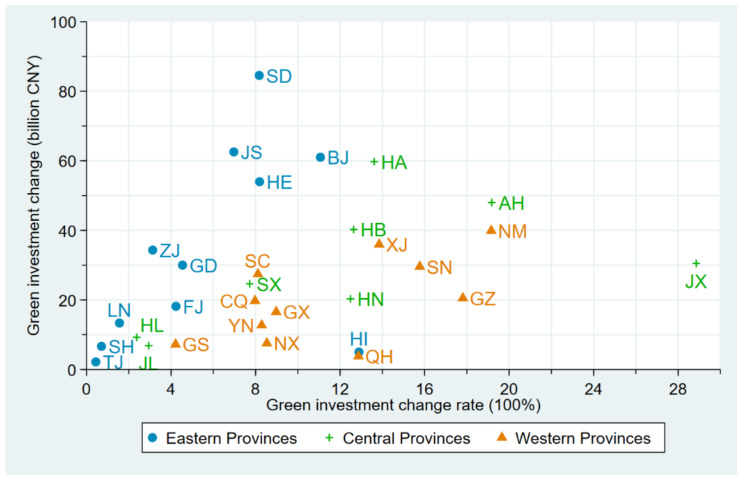
Provincial green investment change and green investment change rate in China during 2002–2017.

Figure 2 shows the rate of change and the amount of change in green investment for 30 provinces in China from 2002 to 2017 in horizontal and vertical coordinates, respectively, and we use three colors and symbols to distinguish between eastern, central, and western provinces. In terms of provincial green investment growth rates (horizontal coordinates), most provinces showed significant growth in green investment from 2002–2017. It is worth noting that 12 of the 30 provinces (40%) had more than 10 times green investment growth, and in particular, JX had green investment growth of 28.86 times, which was mainly due to the especially high priority given to environmental pollution control by the Chinese government during that period. Among the three regions, the green investment growth rates in central and western were significantly higher than those in eastern. More specifically, the provincial averages of green investment growth rates in the east, central and west during the period were 5.63 times, 12.48 times and 11.41 times, respectively. Both central and west had 5 provinces with green investment growth over 10 times, while only 2 provinces, out of 11, in the east showed such dramatic growth. It is noteworthy that 2 provinces, SH and TJ, in the east showed green investment growth less than 1 times with 0.71 and 0.44, respectively. In terms of the amount of provincial green investment growth (vertical coordinate), the amount of green investment growth in the east and central was significantly higher than that in the west from 2002–2017. The provincial average amount of green investment growth in the east, central, and west was 33.80 billion CNY, 29.94 billion CNY and 20.09 billion CNY, respectively. Many policies might have driven the changes, such as the central government strengthening the supervision of local environmental governance and paying attention to the environmental governance performance in the promotion of officials.

### 4.2. National Economic Growth Effect (NEG)

**Figure 3:**
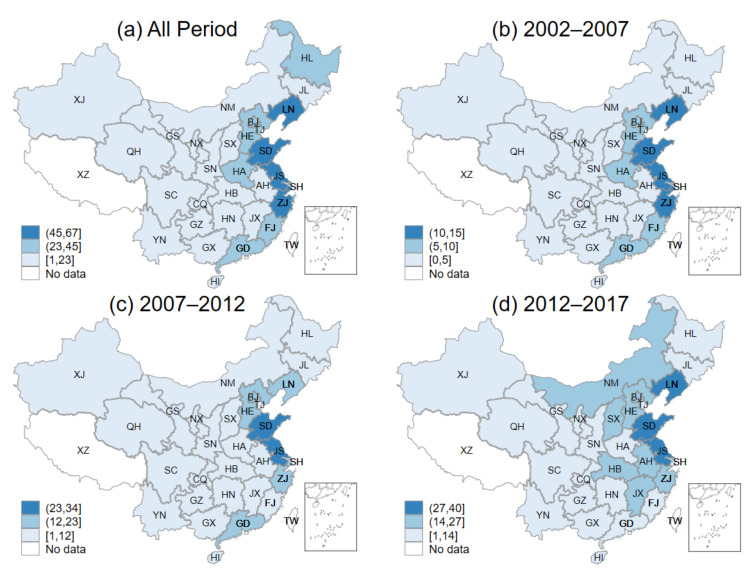
Provincial national economic growth effect: (**a**) all periods; (**b**) 2002–2007; (**c**) 2007–2012; (**d**) 2012–2017.

Figure 3a–d indicate the *NEG* of 30 provinces for the whole period and three periods, respectively. We have equally divided the high, medium, and low ranges according to the maximum and minimum values of *NEG* of each province and marked them with three colors. From Figure 3a, it can be found that the values of *NEG* of all provinces were positive for the whole period, indicating that the national economic growth plays a driving role in the green investment of each province. However, there were large regional differences, with the *NEG* of the eastern provinces substantially higher than that of the central and western provinces. Specifically, there were 5 high *NEG* and 5 medium *NEG* provinces among 11 eastern provinces. By contrast, there was no province with high *NEG* and only 2 with medium *NEG* when considering all 19 western and central provinces. The main reason for the large difference in *NEG* was that the eastern green investment scale was relatively larger than that of the central and western regions throughout the period.

Figure 3b–d represent the provincial *NEG* for the three periods, which are 2002–2007, 2007–2012, and 2012–2017, respectively. The figures clearly state that there were large regional differences in the role of national economic growth in driving provincial green investment in the first two periods (2002–2007 and 2007–2012), and the regional differences began to narrow in the third period (2012–2017). More specifically, the *NEG* of 5 provinces in the eastern region could be categorized as high in the first two periods, while only 1 province in the central region, HA, can be categorized as middle. Meanwhile, none of them was in the high *NEG* category, and all 11 provinces in the west are with low *NEG*. In the third period (2013–2017), although only 3 provinces in the country were with high *NEG*, 8 provinces have *NEG* in the middle category. During 2013–2017, *NEG* indexed of a number of provinces successfully changed from low to medium. Five provinces, NM in the west and SX, HB, AH, and JX in the center, were in the medium *NEG* range. We believed that the main reason for the moderating regional differences was that the previously high *NEG* eastern provinces had difficulty in maintaining high growth rates due to the growth of green investment already reached a much larger scale, resulting in the *NEG* of the central and western starting to gradually converge to that of the east.

For all provinces, the national GDP growth rate and national green investment growth rate are constant, so the differences between *NEG* and the national green investment structure effect (*NIS*) are consistent across provinces. More specifically, the provincial *NIS* shares the same characteristics as the *NEG* except for the size, so there is no need to analyze the *NIS* of each province separately in this study.

### 4.3. Homothetic Regional Green Investment Competition Effect (HRIC)

**Figure 4:**
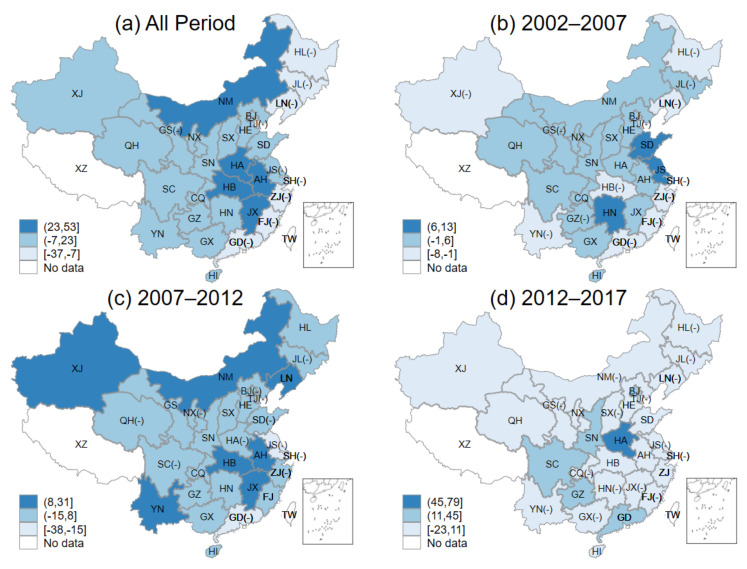
Provincial homothetic regional green investment competition effect: (**a**) all periods; (**b**) 2002–2007; (**c**) 2007–2012; (**d**) 2012–2017.

Figure 4a–d shows the NCE of 30 provinces for the entire period and separately three periods, respectively. Unlike the *NEG* and *NIS*, when the green investment growth rate of a province is lower than the national green investment growth rate, the comparative advantage of that province is negative, resulting in a negative *HRIC*. For this reason, we not only use different colors to classify the *HRIC* of each province into three ranges of low, medium and high but also mark the negative *HRIC* provinces using “(-)” symbols in the figure. A negative *HRIC* represents that the competition effect inhibits green investment from growing.

From Figure 4a, throughout the period, the *HRIC* of central provinces was ahead of the eastern and western. Most eastern provinces were with negative *HRIC*, which means that the competition effect inhibited green investment growth. More specifically, 4 out of 8 central provinces were in the high range of *HRIC* throughout the period, and 17 out of 19 central and western provinces were with medium or high *HRIC* except for HL and JL. 10 provinces in total had negative *HRIC*, accounting for 1/3 of the total amounts of provinces, and 7 out of 11 eastern provinces were with negative *HRIC*. We believe the main reason for this phenomenon was the comparative advantage of green investment of central and western provinces was much larger than that of eastern provinces from 2002 to 2017.

Figure 4b–d show the provincial *HRIC*s in the three periods 2002–2007, 2007–2012, and 2012–2017, respectively. The figures indicate that the *HRIC* of most provinces in these periods showed a general trend of “low/medium-medium/high-low”. More specifically, during the first period, 2002–2007, 27 out of 30 provinces were with medium or low *HRIC*, and only 3 provinces, which are SD, JS, and HN, were with high *HRIC*. During the second period, 2007–2012, 27 provinces were with medium or high *HRIC*, and only three provinces, JS, SH and GD, were with low *HRIC*. During the third period, 2013–2017, 24 provinces were with low *HRIC*, while HA was the only province with high *HRIC*, and only 5 provinces were in the medium range of *HRIC*. We can conclude that, in general, most of the provinces in the country had small regional differences in these three periods.

### 4.4. Regional Green Investment Allocation Effect (RIA)

**Figure 5:**
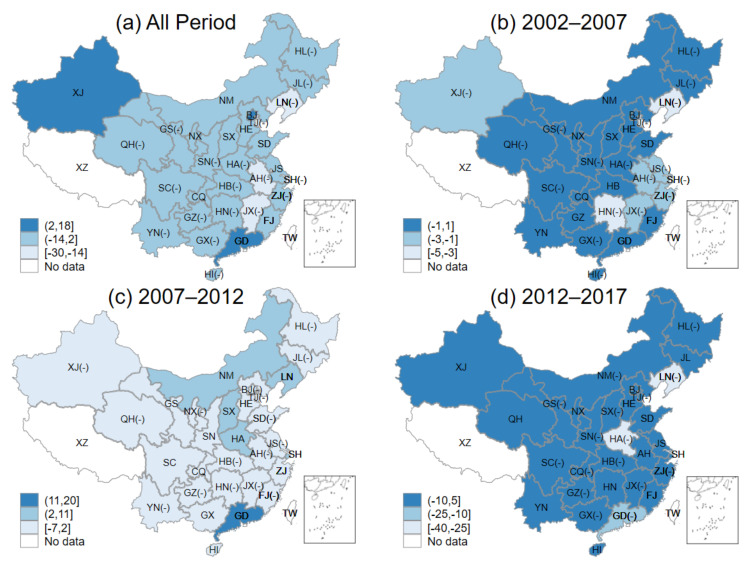
Provincial regional green investment allocation effect: (**a**) all periods; (**b**) 2002–2007; (**c**) 2007–2012; (**d**) 2012–2017.

Figure 5a–d shows the *RIA* of 30 provinces for the entire period and separately three periods, respectively. Since both value of specialization and comparative advantage can be negative, the provincial *RIA* can also be negative, and we have labelled in the same way as we did in the part of *HRIC*. *RIA* reflects whether provinces are specializing in green investments to their advantage or not, i.e., reflecting the impact of provincial green investment allocation effects on green investment changes.

In Figure 5a, it is clear that the green investment allocation effect inhibited provincial green investment growth for most provinces throughout the time period. Although most provinces were with medium *RIA*, given the fact that the range of medium-range was from −143 billion CNY to 13 billion CNY, most provinces still had negative *RIA*. More specifically, 19 out of 30 provinces were with negative *RIA* and only 3 provinces in the country, XJ, BJ, and GD, were with high *RIA*.

Figure 5b–d show the provincial *RIA* in the three periods of 2002–2007, 2007–2012, and 2012–2017, respectively. In general, the *RIA* in most of the provinces indicated a trend of “high-medium-high”. During the first period, 2002–2007, 21 provinces were with high *RIA* while, in the second stage, 2007–2012, 24 provinces could be categorized as low *RIA*, and GD was the only province with high *RIA*. Meanwhile, 5 provinces, NM, LN, SX, HA and SH, were with medium *RIA*. During the third period, 2013–2017, 27 provinces were with high *RIA*, except for LN, HA, and GD with low or medium *RIA*. The intervals of the trends in each time period were (−1, 1], [−7, 2], and (−10, 5], respectively. It suggested that the effect of *RIA* was weak in most of the provinces in these three periods.

### 4.5. Specialization and Comparative Advantage

**Figure 6:**
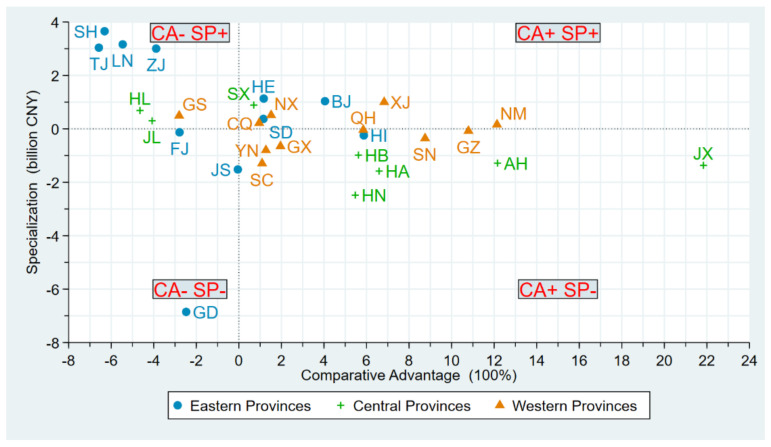
Provincial green investment specialization and comparative advantage in China during 2002–2017.

Given the fact that *RIA* is the product of Specialization (SP) and Comparative Advantage (CA), to further analyze *RIA*, we have created four quadrants with CA and SP for each province (see Figure 6). As stated in the methodology section, if a province is in the first quadrant or the third quadrant, meaning both CA and SP have the same sign, positive or negative, its *RIA* is positive. When a province is in the second quadrant or the fourth quadrant, meaning CA and SP have different sign, the *RIA* of the province is negative. In Figure 6, it is clear that most of the provinces were located in the second and fourth quadrants, suggesting the *RIA* of these provinces was negative. More specifically, in the 4 eastern provinces, SH, TJ, LN, and ZJ, although they had better green investment share than the entire country’s level, green investment growth was relatively disadvantageous, resulting in these 4 eastern provinces having negative *RIA*. For 16 central and western provinces, green investment growth was comparatively advantageous. However, only 5 of these 16 provinces had a high green investment share than the national level, resulting in positive *RIA* for these 5 provinces, leaving 11 central and western provinces with negative *RIA*.

## 5. Conclusions

Green investment is the foundation and important determinant for environmental governance. However, few studies have paid special attention to the driving factors of green investment changes in Chinese provinces. We use HSSA to decompose the changes of green investment of 30 provinces in China from 2002 to 2017 into four factors: national economic growth effect, national green investment structure effect, homothetic regional green investment competition effect and regional green investment allocation effect. According to three periods of 2002–2007, 2007–2012, and 2012–2017, the differences in the changes of each effect in the eastern, central and western regions were analyzed. The main conclusions are as follows:

(1) Given the change in green investment, from 2002 to 2017, China’s green investment showed a significant upward trend, and the proportion of China’s green investment to GDP maintained steady growth. Although green investment had been increasing in all provinces of the three major regions from 2002 to 2017, the growth rate of green investment in the central and western region was significantly higher than that in the eastern region. The amount of green investment growth in the eastern and central regions was significantly higher than that in the western region.

(2) In terms of the National economic growth effect (*NEG*), all 30 provinces had positive *NEG* from 2002 to 2017, but there were large regional differences, with the eastern provinces significantly outperforming the central and western provinces. The regional differences were relatively large in the first two periods (2002–2007 and 2007–2012) and began to shrink in the third period (2012–2017).

(3) From the perspective of the Homothetic Regional Green Investment Competition Effect (*HRIC*), the central provinces were ahead of the eastern and western regions from 2002 to 2017, and relatively many eastern provinces were with negative *HRIC*. During these three periods, the *HRIC* of most provinces showed a trend of “low/medium–medium/high–low”.

(4) In terms of the Regional Green Investment Allocation Effect (*RIA*), during 2002–2017, the allocation effect of green investment inhibited provincial green investment growth in most provinces. Although most of the provinces showed a “high–low–high” trend in *RIA* during these three periods, the effect of *RIA* could be considered weak. In terms of specialization and comparative advantage, the main reason why the allocation effect inhibited growth in most provinces was that while most central and western regions had comparative advantages on green investment growth, these regions did not specialize in green investment at the same time.

This study also provides several implications for policy-makers. First, our study suggests that it is necessary to coordinate the growth of green investment across different regions to improve the utilization efficiency of green investment. In central and western regions, although the growth rate of green investment is fast, the total amount is still insufficient. The governments in these regions should further increase environmental investment and strictly implement regulatory policies to restrict the negative externality of green investment shortage. In the eastern region, the growth rate of green investment has relatively slowed down. Governments in the eastern region could guide and encourage more private green investment and further expand the growth rate of green investment. Second, it is necessary for the central government to establish an ecological compensation mechanism to adjust the interest relationship between the parties concerned in ecological environment protection and construction. Green investment has strong positive externalities, and it may damage the investment incentives of some regions, especially the underdeveloped regions. As the beneficiary, the eastern region is necessary to compensate for the green investment in the central and western regions to stimulate the growth of the green investment.

However, some limitations should be noted. First, by using the SSA decomposition method, we obtained the results of various types of effects. However, we can only qualitatively explain the reasons for the differences between the effects and lack a rigorous causal inference. Second, provinces were grouped and discussed using eastern, central, and western, although this made it easy for us to find out the differences in the effects of each region. However, this grouping method was difficult to help us observe the overall provincial differences.

## Data Availability

The data presented in this study are available on request from the corresponding author.
